# Self-Compassion and Smartphone Addiction Tendency Among College Students: The Chain-Mediating Effect of Self-Concept Clarity and Experiential Avoidance

**DOI:** 10.3390/bs15040512

**Published:** 2025-04-11

**Authors:** Yin Qiu, Shaoying Gong, Yang Yang, Jing Wang, Liping Tan

**Affiliations:** 1 Key Laboratory of Adolescent Cyberpsychology and Behavior (CCNU), Ministry of Education, Wuhan 430079, China; qiuyin@mails.ccnu.edu.cn (Y.Q.); yangyang@huanghuai.edu.cn (Y.Y.); wangjingpsy@mails.ccnu.edu.cn (J.W.); tanlip@mails.ccnu.edu.cn (L.T.); 2 Key Laboratory of Human Development and Mental Health of Hubei Province, School of Psychology, Central China Normal University, Wuhan 430079, China; 3 Mental Health Education Center, Wuhan College, Wuhan 430212, China; 4 Mental Health Education Center, Huanghuai University, Zhumadian 463000, China

**Keywords:** self-compassion, smartphone addiction tendency, self-concept clarity, experiential avoidance

## Abstract

Smartphone addiction has emerged as a pressing public health issue in recent years, which negatively impacts university students’ academic performance, physical and mental health, and social functioning. Therefore, it is crucial to explore the significant factors related to smartphone addiction. While previous research has suggested a potential link between self-compassion and problematic internet or smartphone use, studies specifically examining the relationship between self-compassion and smartphone addiction tendency remain limited. From a positive psychology perspective, this study aims to explore the relationship between self-compassion and smartphone addiction tendency, as well as its internal mechanism. A total of 641 Chinese college students were recruited to complete online questionnaires assessing their self-compassion, self-concept clarity, experiential avoidance, and smartphone addiction tendency. The results show that self-compassion not only directly and negatively predicts smartphone addiction tendency, but also indirectly predicts it through the independent mediating effects of self-concept clarity and experiential avoidance. Additionally, there is a chain-mediating effect of self-concept clarity and experiential avoidance. This study provides a new perspective for the prevention and intervention of smartphone addiction tendency among college students.

## 1. Introduction

With the rapid development of mobile internet technology, smartphones are playing an increasingly important role in the lives of modern people. According to the China Internet Network Information Center (CNNIC, 2024), as of June 2024, 99.7% of internet users in China accessed the internet using smartphones. Smartphones have become an integral part of peoples’ lives due to their accessibility and convenience, along with personalized content and functionality that cater to diverse user needs (Q. Liu et al., 2017). However, this pervasive usage has led to mounting concerns about the risk of smartphone addiction.

Smartphone addiction, also known as smartphone dependence or problematic smartphone use, is a behavioral addiction characterized by excessive and uncontrollable smartphone use, leading to significant impairments in psychological, behavioral, and social functioning (Barnes et al., 2019; Q. Liu et al., 2020; Samaha & Hawi, 2016). A meta-analysis indicated a significant upward trend in smartphone addiction among Chinese college students in recent years (Peng et al., 2022). This addiction can negatively affect students’ academic performance, physical and mental health, and their social functioning (Olson et al., 2022; H. Wei et al., 2023). A better understanding of the antecedents and potential mechanisms of college students’ smartphone addiction tendency is crucial for the development of effective prevention and intervention strategies.

Previous studies have primarily focused on individual and environmental risk factors for smartphone addiction tendency, such as negative parenting styles (Y. Yang et al., 2024), adverse childhood experiences (Lin et al., 2024), and mental health issues (Busch & McCarthy, 2021). In contrast to traditional psychology’s focus on problems and weaknesses, positive psychology emphasizes identifying protective factors and enhancing individuals’ well-being (Seligman & Csikszentmihalyi, 2000). As a result, researchers have increasingly turned to positive psychology to explore the protective factors that could prevent or reduce internet addiction (Khazaei et al., 2017).

From a positive psychology perspective, smartphone addiction is not only a result of environmental risk factors, but also serves as a coping strategy for individuals lacking psychological resources. According to the Conservation of Resources theory (COR, Hobfoll, 2011), individuals with limited psychological resources may turn to maladaptive coping strategies, such as excessive smartphone or internet use (Hao et al., 2023; Wang et al., 2024). Self-compassion, recognized as a crucial protective psychological resource (T. Lee et al., 2022; Tiwari et al., 2020), has been consistently found to be negatively correlated with various forms of addictive behaviors (Iskender & Akin, 2011; Geng et al., 2022; Q.-Q. Liu et al., 2020; Phelps et al., 2018). Unlike self-esteem, which often relies on external validation or personal achievements, self-compassion involves treating oneself with kindness and understanding in the face of setbacks and distress (Neff, 2003). This approach promotes a more stable sense of self-worth (Neff & Vonk, 2009), enabling individuals to better integrate challenging experiences and regulate emotions more effectively. More importantly, self-compassion is highly malleable and can be cultivated through both brief and long-term interventions (Miyagawa, 2024; Neff, 2023).

Researchers have found that self-compassion is negatively associated with various forms of addictive and problematic behaviors (Iskender & Akin, 2011; Geng et al., 2022; Q.-Q. Liu et al., 2020; Phelps et al., 2018). However, limited studies have examined the relationship between self-compassion and smartphone addiction tendency, as well as the underlying mechanisms. Therefore, this study aims to explore how self-compassion influences smartphone addiction tendency among college students and investigate the roles of self-concept clarity and experiential avoidance in this relationship.

### 1.1. Self-Compassion and Smartphone Addiction Tendency

Self-compassion is a way for individuals to treat themselves in the face of failures, inadequacies, and suffering (Neff, 2003). It entails three basic components: (a) self-kindness—responding to one’s flaws and suffering with kindness or understanding rather than harsh self-judgment; (b) common humanity—recognizing imperfection and failure as universal human experiences, fostering connection rather than isolation; and (c) mindfulness—maintaining a balanced awareness of present thoughts and emotions, and avoiding excessive immersion in negative feelings or thoughts (Neff, 2023). These components interact with each other to collectively form a dynamic system of collaborative synergy (Dreisoerner et al., 2021).

According to the social mentality theory proposed by Gilbert (2009), an individual’s affect regulation system can be divided into three main categories based on their functional differences: the threat protection system, drive system, and contentment system. Self-compassion can activate the contentment system, fostering a sense of inner calm and security, similar to the feeling of having a warm, supportive, and empathetic friend by your side (Gilbert, 2009). A meta-analysis found a positive correlation between self-compassion and various forms of individual well-being (Zessin et al., 2015). Longitudinal studies have suggested that self-compassion can enhance well-being by fulfilling basic psychological needs (Gunnell et al., 2017; X. Y. Liu, 2022). This relationship is particularly relevant when considering the compensatory internet use theory (Kardefelt-Winther, 2014), which suggests that individuals often turn to the internet as a means of coping with unmet psychological needs and real-life stress. Self-compassion may reduce this reliance on the virtual world by fulfilling basic psychological needs in real life and promoting positive psychological states, thereby decreasing the risk of smartphone addiction tendency.

Furthermore, empirical studies have indicated that individuals with high self-compassion demonstrate more effective emotion regulation strategies, adopt more adaptive coping strategies, and possess richer psychological resources (Bakker et al., 2019; Diedrich et al., 2014; Ewert et al., 2021; Y. Yang et al., 2016). These factors contribute to a reduced tendency toward smartphone addiction. Several studies have further supported the protective role of self-compassion against addiction. For example, Iskender and Akin (2011) found a negative correlation between self-compassion and internet addiction among college students. Similarly, P. Wei (2024) found that self-compassion reduces social media addiction, with gratitude serving as a mediator. More directly, Geng et al. (2022) found that self-compassion is negatively correlated with problematic smartphone use among adolescents. Based on these findings, we speculate that self-compassion might be negatively associated with smartphone addiction tendency among college students.

### 1.2. Self-Concept Clarity as a Mediator

Self-concept clarity is defined as the extent to which the contents of an individual’s self-concept are clearly and confidently defined, with internal consistency and temporal stability (Campbell et al., 1996). Self-compassionate individuals are theorized to approach their failures or personal inadequacies with warmth and kindness, rather than harsh judgment (Neff, 2003). This positive self-attitude reduces defensiveness toward negative aspects of the self, allowing them to be integrated into one’s overall self-concept (Thomas et al., 2013). This balanced perspective enhances both the integrity and clarity of the self-concept. Furthermore, individuals with high self-compassion do not construct their self-concept based on social comparisons or external evaluations (Neff & Vonk, 2009). Instead, they focus on feelings of compassion toward themselves and the recognition of their common humanity, which reduces the distortions in their self-concept and enhances its stability (Neff, 2003). Previous studies have indicated that mindfulness, as a core component of self-compassion, is positively correlated with self-concept clarity (Hanley & Garland, 2017; F. Yang & Oshio, 2024). A recent experimental study further demonstrated that inducing self-compassion enhances individuals’ self-concept clarity, with moderate-to-large effect sizes (Miyagawa, 2024). Thus, self-compassion may positively associate with self-concept clarity.

Self-concept clarity has emerged as a significant factor that may be related to smartphone addiction tendency. Self-regulation theory suggests that the self-concept plays a critical role in self-regulation. Specifically, individuals with low self-concept clarity have difficulty processing and integrating self-relevant information effectively, which leads them to rely more on external cues for decision-making and results in poorer self-regulation abilities (Setterlund & Niedenthal, 1993). According to self-regulation theory, individuals with low self-concept clarity lack sufficient self-control (T. Jiang et al., 2023), making them more prone to developing addictive tendencies when using smartphones. Additionally, individuals with low self-concept clarity may experience uncertainty about themselves. According to the uncertainty–identity theory, feeling uncertain about oneself triggers aversion and discomfort, which drives individuals to take actions to reduce this uncertainty (Hogg, 2012). Individuals with low self-concept clarity tend to use smartphones more frequently to seek feedback about their self-concept through information searching and online social interaction (Quinones & Kakabadse, 2015). These behaviors increase the risk of internet or smartphone addiction (Israelashvili et al., 2012). Previous studies have also demonstrated a negative association between self-concept clarity and problematic smartphone use (Servidio et al., 2021; Xie et al., 2024). Considering that self-compassion may be associated with self-concept clarity, and self-concept clarity may in turn be negatively correlated with smartphone addiction tendency, it is plausible that self-concept clarity could mediate the relationship between self-compassion and smartphone addiction tendency.

### 1.3. Experiential Avoidance as a Mediator

Experiential avoidance refers to an unwillingness to experience unwanted internal thoughts, feelings, and sensations. Individuals negatively evaluate these experiences and actively avoid, control, or escape them, along with the physiological arousal that accompanies them (Goodman et al., 2018). Experiential avoidance can be an adaptive short-term emotional regulation strategy. However, when it becomes an inflexible behavioral pattern, it can result in numerous negative outcomes (García-Oliva & Piqueras, 2016).

Self-compassion may reduce individuals’ experiential avoidance. Research has shown a negative correlation between self-compassion and experiential avoidance (Ewert et al., 2021). Additionally, Yela et al. (2022) found that interventions targeting self-compassion can significantly reduce experiential avoidance. Given that high emotional intensity and the cognitive appraisal of emotions as threats increase the likelihood of experiential avoidance (Goodman et al., 2018), self-compassion may reduce experiential avoidance in two ways: First, according to the social mentality theory, compassion is part of a prosocial motivational system that can alleviate suffering through warmth, safeness, and connection (Gilbert, 2009, 2019), thereby reducing experiential avoidance. Second, self-compassion encourages individuals to approach negative emotions with openness and acceptance, which helps reappraise these emotions as less threatening and fosters greater emotional tolerance. Together, these processes may reduce the reliance on experiential avoidance as a coping strategy. Thus, self-compassion may negatively predict experiential avoidance.

On the other hand, experiential avoidance may increase smartphone addiction tendency. According to the experiential avoidance model, individuals with high experiential avoidance often engage in various maladaptive behaviors to avoid their inner emotional experiences (Chapman et al., 2006). For individuals with high experiential avoidance, smartphones serve as a new tool to avoid distressing emotions through engaging content and online social interactions (Xiong et al., 2023), which further increases their risk of smartphone addiction. In addition, according to the limited self-control model, the psychological resources people use for self-regulation are limited (Muraven & Baumeister, 2000). Individuals who rely on experiential avoidance devote considerable time, attention, and energy to managing negative emotions. This depletion of self-regulatory resources makes it more difficult for individuals to resist the impulsive use of smartphones, ultimately contributing to the development of smartphone addiction tendency (Z. Jiang & Zhao, 2017; H. Wei et al., 2023). For instance, previous studies have shown that college students with high experiential avoidance are more likely to develop addictions to social media, the internet, and smartphones (Chou et al., 2017; Faghani et al., 2020; Zhang & Wang, 2022). Therefore, we infer that experiential avoidance, as a proximal influencing factor of addictive behaviors, may serve as another mediating variable between self-compassion and smartphone addiction tendency.

### 1.4. The Chain Mediation of Self-Concept Clarity and Experiential Avoidance

Self-concept clarity is closely related to experiential avoidance. Numerous self-regulation theories emphasize the central role of the self-concept in emotional and behavioral regulation, suggest that disruptions to one’s self-concept, particularly self-doubt or uncertainty, impair self-regulation (Duval & Wicklund, 1972; Higgins, 1987; Light, 2017). Therefore, self-concept clarity is a crucial resource for self-regulation. Individuals with low self-concept clarity struggle to accurately identify and understand their emotions, which limits their ability to engage in adaptive emotional regulation. Since the experiential avoidance model highlights that a lack of effective emotional regulation strategies contributes to experiential avoidance (Chapman et al., 2006), self-concept clarity may help reduce experiential avoidance by promoting adaptive emotional regulation.

Previous research has shown that identity confusion predicts higher levels of experiential avoidance (Berzonsky & Kinney, 2019). Individuals with high self-concept clarity tend to adopt adaptive coping strategies to manage emotional distress, rather than avoid negative emotions (Ren et al., 2022). In contrast, individuals with low self-concept clarity often struggle with emotional and identity ambiguity, and tend to engage in maladaptive coping behaviors, such as avoidance, withdrawal, or a denial of problems (Smith et al., 1996). Recently, a daily diary study extended this negative association from between individuals to within individuals, revealing that on days when individuals have higher self-concept clarity, they are less likely to use emotional disengagement strategies, such as suppression (Landa, 2021).

More importantly, according to the Interaction of Person–Affect–Cognition–Execution (I-PACE) Model for internet addiction, individual traits influence executive function and inhibitory control through specific affective and cognitive responses, ultimately contributing to internet addiction (Brand et al., 2016). Self-concept clarity is a malleable individual characteristic (Hertel, 2017), while experiential avoidance is a maladaptive coping strategy. It is possible that self-compassion influences smartphone addiction tendency through the mediation of self-concept clarity and experiential avoidance. Based on the aforementioned theories and empirical evidence, we propose that self-concept clarity and experiential avoidance play a chain-mediating role in the relationship between self-compassion and smartphone addiction tendency.

### 1.5. Present Study

While numerous studies have examined the influence of self-compassion on well-being and psychopathology, less attention has been paid to its relationship with addictive behaviors, particularly smartphone addiction. Therefore, this study aims to explore the impact of self-compassion on smartphone addiction tendency among college students. The following hypotheses are proposed:

**H1.**  *Self-compassion is negatively associated with smartphone addiction tendency.*

**H2.**  *Self-concept clarity mediates the association between self-compassion and smartphone addiction tendency.*

**H3.**  *Experiential avoidance mediates the association between self-compassion and smartphone addiction tendency.*

**H4.**  *Self-compassion negatively correlates with smartphone addiction tendency through the chain mediation of self-concept clarity and experiential avoidance.*

## 2. Materials and Methods

### 2.1. Participants

According to the a priori sample size calculator for structural equation modeling, with a statistical power level of 0.8, considering 4 latent variables, 26 observed variables, and an anticipated medium effect size, the recommended minimum sample size is 288 (Memon et al., 2020; Soper, 2020).

Data collection was conducted through an online survey on the Questionnaire Star platform. The questionnaire link was distributed to college student recruitment groups from provinces including Hubei, Henan, and Shanxi in China. Participants received standardized instructions outlining the survey’s purpose, guidelines for responding to the questions, and the principle of voluntariness. A total of 680 college students agreed to participate in this study and completed the questionnaire. After removing invalid questionnaires (those in which participants provided identical or nearly identical responses to all items), a total of 641 participants were retained, yielding a validity rate of 94%. Among them, 443 (69.11%) were female (*M*_age_ = 19.78, *SD* = 1.49, age range = 17–24 years), 355 were freshmen, 127 were sophomores, 77 were juniors, and 82 were seniors. Additionally, 238 participants were from urban areas, and 403 were from rural areas.

### 2.2. Measures

#### 2.2.1. Self-Compassion

Self-compassion was assessed using the Chinese version of the Self-Compassion Scale (SCS; Gong et al., 2014), which was translated and revised from the original version developed by Neff (2003). This version of the scale has been widely used among Chinese college students and adolescents, showing good reliability and validity (Chen et al., 2022; Gong et al., 2014; Pi et al., 2024). The revised scale consists of 12 items, with 7 items scored positively and 5 items scored inversely. It includes three dimensions: self-kindness (sample item: “I’m intolerant and impatient towards those aspects of my personality I don’t like”), common humanity (sample item: “When I fail at something that’s important to me, I tend to feel alone in my failure”), and mindfulness (sample item: “When something upsets me, I try to keep my emotions in balance”). Each item was rated on a 5-point Likert scale from 1 (almost never) to 5 (almost always). A higher total score indicated a higher level of self-compassion. In this study, confirmatory factor analysis demonstrated good fit for the three-factor structure: χ^2^/*df* = 1.95, RMSEA = 0.04, CFI = 0.95, TLI = 0.94, and SRMR = 0.04. The Cronbach’s alpha coefficient for the scale was 0.82.

#### 2.2.2. Self-Concept Clarity

Self-concept clarity was measured using the Self-Concept Clarity Scale (SCCS; Campbell et al., 1996; Niu et al., 2016). The scale consists of 12 items (sample item: “I have a clear sense of who I am and what I stand for”) using a 5-point Likert scale (1 representing “strongly disagree” and 5 representing “strongly agree”). Confirmatory factor analysis supported the unidimensional structure with good fit: χ^2^/*df* = 2.69, RMSEA = 0.05, CFI = 0.94, TLI = 0.92, and SRMR = 0.04. Higher scores indicated greater self-concept clarity. The Cronbach’s alpha coefficient for the scale was 0.84 in this study.

#### 2.2.3. Experiential Avoidance

This study assessed experiential avoidance using the Acceptance and Action Questionnaire (AAQ-II; Bond et al., 2011). The scale consists of 7 items (sample item: “I worry about not being able to control my worries and feelings”). Each item was rated on a 7-point Likert scale from 1 (never true) to 7 (always true). Confirmatory factor analysis supported the unidimensional structure with good fit: χ^2^/*df* = 3.81, RMSEA = 0.07, CFI = 0.97, TLI = 0.96, and SRMR = 0.03. Higher total scores reflected greater experiential avoidance. In this study, the Cronbach’s alpha coefficient for the scale was 0.92.

#### 2.2.4. Smartphone Addiction Tendency

Smartphone addiction tendency was assessed using the Chinese version of the Mobile Phone Addiction Tendency Scale (MPATS; Xiong et al., 2012). The scale consists of 16 items and includes four dimensions: withdrawal symptoms (sample item: “If I don’t use my phone for a long time, I feel uncomfortable”), salience (sample item: “If I go too long without using my phone, I start to feel uneasy”), social comfort (sample item: “I’d rather chat on my phone than talk face-to-face”), and mood changes (sample item: “I’m often afraid that my phone will shut off automatically”). Responses were given on a 5-point Likert scale ranging from 1 (strongly disagree) to 5 (strongly agree). The MPATS has been widely used in studies involving Chinese university students, demonstrating good psychometric properties, as well as consistent reliability and validity (Ling et al., 2025; Xu et al., 2021; H. Wei et al., 2023). Confirmatory factor analysis supported the four-factor structure with acceptable fit: χ^2^/*df* = 4.01, RMSEA = 0.07, CFI = 0.93, TLI = 0.91, and SRMR = 0.05. Higher scores indicated a higher degree of smartphone addiction tendency. The Cronbach’s alpha coefficient was 0.94 in this study.

### 2.3. Procedure and Data Analyses

Data analysis proceeded in two phases. First, descriptive statistics and correlations were computed using SPSS 25.0. Subsequently, hypotheses were tested using structural equation modeling in Mplus 8.0. Robust maximum likelihood estimation was employed. Mediation effects were examined using the bootstrap method with 5000 resamples to generate 95% confidence intervals (CIs). Effects were considered significant if the CIs did not include zero (Kline, 2018).

Model fit was evaluated using multiple indices, including the chi-square (χ^2^), Comparative Fit Index (CFI), Tucker–Lewis Index (TLI), Root Mean Square Error of Approximation (RMSEA), and Standardized Root Mean Square Residual (SRMR). Model fit was considered acceptable when SRMR and RMSEA values were ≤ 0.08 and CFI and TLI values were ≥ 0.90 (Bentler & Bonett, 1980; Browne & Cudeck, 1992). A good model fit was indicated when RMSEA was < 0.05 and CFI and TLI exceeded 0.95.

## 3. Results

### 3.1. Discriminant Validity and Common Method Bias Test

We first constructed Model 1, treating self-compassion, self-concept clarity, experiential avoidance, and smartphone addiction tendency as four independent factors. Next, we gradually merged the constructs to form Models 2, 3, and 4, and compared these models with Model 1. As shown in Table 1, the four-factor model demonstrated a significantly better fit than the alternative models. The TLI and CFI of Model 1 decreased by 0.05 to 0.20 compared to the other competing models, exceeding the 0.03 threshold. Similarly, the RMSEA increased by 0.03 to 0.08, surpassing the 0.01 standard (Wen et al., 2018). These results indicate that the four variables in this study exhibited good discriminant validity. Additionally, the factor loadings for each factor ranged from 0.67 to 0.90 (*ps* < 0.001), and the average variance extracted (AVE) for each factor was greater than 0.50, with the composite reliability (CR) for each factor exceeding 0.75. These values met the standards for convergent validity (Wu, 2013), indicating that the main variables in this study demonstrated a high convergent validity. Furthermore, the correlations between the constructs were all smaller than the square roots of their respective AVE values, further supporting the model’s good discriminant validity.

To examine whether common method bias exists in this study, we constructed a five-factor model (*χ*^2^/*df* = 4.64, CFI = 0.94, TLI = 0.93, RMSEA = 0.07, and SRMR = 0.05) by adding a latent factor representing the potential common method bias to the original four-factor model. A comparison of the model fit indices before and after controlling for the latent factor indicated that all the changes were within 0.02, suggesting that common method bias was not a significant concern in this study (Podsakoff et al., 2003).

### 3.2. Descriptive Statistics and Correlation Analysis of Variables

Since the smartphone addiction tendency differed by grade, we used a partial correlation analysis to control for this variable. The results show that self-compassion was positively correlated with self-concept clarity and negatively correlated with experiential avoidance and smartphone addiction tendency. Additionally, self-concept clarity was negatively correlated with both experiential avoidance and smartphone addiction tendency. Experiential avoidance and smartphone addiction tendency were positively correlated with each other (see Table 2).

### 3.3. Mediation Effect Analysis

**Figure 1:**
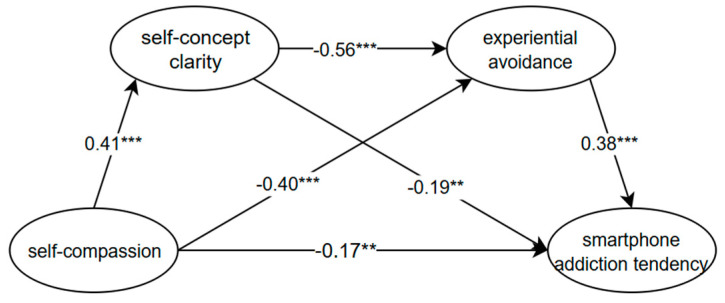
Chain-mediating effect of self-concept clarity and experiential avoidance. Note: ** *p* < 0.01, *** *p*< 0.001.

We constructed a chained mediation model with self-compassion as the independent variable, smartphone addiction tendency as the dependent variable, and self-concept clarity and experiential avoidance as the mediators, while controlling for the grade. The model showed an acceptable fit to the data (χ^2^(129) = 567.43, RMSEA = 0.07, CFI = 0.94, TLI = 0.92, SRMR = 0.05). The results, as shown in Figure 1, indicate that self-compassion significantly negatively predicted smartphone addiction tendency (*β* = −0.17, *p* < 0.01) and experiential avoidance (*β* = −0.40, *p* < 0.001), but it significantly positively predicted self-concept clarity (*β* = 0.41, *p* < 0.001). Self-concept clarity significantly negatively predicted experiential avoidance (*β* = −0.56, *p* < 0.001) and smartphone addiction tendency (*β* = −0.19, *p* < 0.01). Experiential avoidance also significantly positively predicted smartphone addiction tendency (*β* = 0.38, *p* < 0.001).

Table 3 presents the results of the bootstrap mediation analysis, revealing the significant independent mediation effect of self-concept clarity (indirect effect = −0.09; 95% CI [−0.16, −0.03]), accounting for 17.69% of the total effect. Experiential avoidance also showed a significant independent mediation effect (indirect effect = −0.15; 95% CI [−0.24, −0.09]), contributing to 30.61% of the total effect. Additionally, the chain mediation effect involving self-concept clarity and experiential avoidance was significant (indirect effect = −0.09; 95% CI [−0.14, −0.05]), accounting for 17.50% of the total effect.

## 4. Discussion

Based on social mentality theory and the I-PACE model, we examined the effect of self-compassion on smartphone addiction tendency. The results show that self-compassion was significantly related to smartphone addiction tendency, with self-concept clarity and experiential avoidance playing mediating roles. These findings not only support existing theories, but also provide new insights into smartphone addiction interventions.

### 4.1. The Effect of Self-Compassion on Smartphone Addiction Tendency

This study demonstrates a negative association between self-compassion and smartphone addiction tendency, supporting Hypothesis 1, which is consistent with previous research (Geng et al., 2022). Previous research has demonstrated that self-compassion is a significant predictor of reduced psychological symptoms (E. E. Lee et al., 2021; MacBeth & Gumley, 2012), with self-compassion-based interventions showing sustained effectiveness for alleviating internalizing problems (Ferrari et al., 2019). Our findings extend the positive effects of self-compassion, confirming its protective role in preventing smartphone addiction.

Self-compassion, as a supportive attitude towards oneself, does not mean going easy on oneself or leading to self-indulgence (Neff, 2023). Instead, individuals with high self-compassion exhibit greater personal initiative (Dundas et al., 2017), a stronger focus on their long-term personal goals, and engage in more adaptive behaviors rather than maladaptive behaviors (Sirois et al., 2019; Wong et al., 2021). Self-compassion enables individuals to build an internal motivational system and to clarify their core values (Hope et al., 2014; Neff, 2023; Neff et al., 2005), and enables them to prioritize activities that contribute to their long-term well-being and personal growth, rather than being tempted by the immediate gratification offered by smartphones. By promoting goal-directed behaviors, self-compassion strengthens resistance to external distractions and reduces reliance on instant gratification, ultimately reducing smartphone addiction tendency.

On the other hand, individuals prone to smartphone addiction may lack an awareness of their behavior; self-compassion provides a supportive inner environment that helps them better recognize and regulate their emotions and actions. Self-compassion promotes individuals’ kindness and understanding toward themselves, encouraging them to adopt a balanced perspective when observing their experiences. This enables them to better recognize and manage their negative emotions and addictive impulses (Beshai et al., 2018; Diedrich et al., 2017; Finlay-Jones, 2023), promoting a healthier engagement with their surroundings and reducing excessive reliance on smartphones. Additionally, self-compassion fosters a sense of common humanity, helping individuals feel more connected to others and reducing the tendency to seek comfort through smartphones in response to loneliness. In contrast, individuals with low self-compassion tend to engage in self-criticism during difficult times, which triggers negative emotions and maladaptive coping strategies (Carona et al., 2022; Eichholz et al., 2020), such as excessive smartphone use.

### 4.2. The Mediating Role of Self-Concept Clarity

The present study finds that self-concept clarity serves as a mediator in the relationship between self-compassion and smartphone addiction tendency, supporting Hypothesis 2.

The core components of self-compassion include self-kindness, common humanity, and mindfulness (Neff, 2023). Self-kindness helps individuals reduce defensiveness toward their flaws and deficiencies, allowing them to view themselves more comprehensively (Miyagawa, 2024). Common humanity views personal suffering as a shared human experience, which can help individuals expand their self-awareness. Mindfulness promotes present-moment engagement, enhances the ability to perceive both internal and external information (Ren et al., 2022), reduces cognitive biases and habitual thought patterns (Vago & Silbersweig, 2012), and supports the development of self-relevant insights. Together, these components of self-compassion contribute to improving self-concept clarity. Previous research has also confirmed the role of self-compassion in enhancing self-concept clarity (Miyagawa, 2024). Conversely, individuals with low self-compassion are more prone to self-judgment, isolation, and over-identification, which undermines their ability to view themselves objectively and reduces their self-concept clarity.

On the other hand, self-concept clarity reduces smartphone addiction tendency, which is consistent with previous research (Servidio et al., 2021). Individuals with high self-concept clarity have a stable and consistent sense of self, allowing them to recognize their emotions, needs, and goals without frequently seeking external validation. On the contrary, individuals with low self-concept clarity prefer online social interactions to seek recognition of their identity and value (Quinones & Kakabadse, 2015), which increases the likelihood of smartphone addiction. Moreover, a clear and confident sense of self enhances self-regulation, while uncertainty about one’s identity undermines self-control and goal pursuit (T. Jiang et al., 2023; Light, 2017). As a result, a high self-concept clarity enables individuals to better regulate their behaviors, including smartphone use. In summary, self-compassion promotes self-concept clarity by fostering self-kindness, common humanity, and mindfulness, which collectively enhance individuals’ ability to maintain a stable and consistent sense of self. In turn, a clearer self-concept helps individuals resist the temptation of excessive smartphone use, thereby reducing smartphone addiction tendency.

### 4.3. The Mediating Role of Experiential Avoidance

This study finds that college students’ self-compassion can negatively predict smartphone addiction tendency through the mediating role of experiential avoidance, which verifies Hypothesis 3.

This result suggests that individuals with high self-compassion are less likely to engage in experiential avoidance behaviors. This aligns with the experiential avoidance model, which emphasizes that individuals often suppress or avoid negative emotions due to low distress tolerance or limited emotion regulation skills (Goodman et al., 2018). By fostering a non-judgmental acceptance of emotions, self-compassion encourages individuals to approach difficult feelings with greater openness, thus reducing reliance on avoidance strategies (Bicaker et al., 2022). Additionally, individuals with high self-compassion tend to possess a broader range of emotion regulation strategies (Finlay-Jones, 2023), further reducing the likelihood of experiential avoidance.

The relationship between experiential avoidance and smartphone addiction tendency is also clearly demonstrated in this study. Since smartphones are easily accessible, they allow individuals to quickly divert their attention, offering short-term relief from negative emotions. This temporary escape reinforces the habit of using smartphones in similar situations. Moreover, attempting to suppress and avoid certain thoughts can increase their frequency (Abramowitz et al., 2001). As a result, using a smartphone to escape from negative emotions may trigger a rebound effect, intensifying those emotions and driving further phone use. This cycle may contribute to the development of smartphone addiction tendency.

This finding expands the application of the experiential avoidance model, which is commonly used to explain phenomena, such as self-harm, suicide, and substance abuse (Chapman et al., 2006). This study suggests that smartphone addiction tendency can also be understood from the perspective of experiential avoidance.

### 4.4. The Chain-Mediating Role of Self-Concept Clarity and Experiential Avoidance

This study also finds that self-concept clarity and experiential avoidance act as chain mediators between self-compassion and smartphone addiction tendency, which proves Hypothesis 4 and supports the I-PACE model.

As aforementioned, self-compassion encourages a kind, balanced, and non-judgmental view of oneself, which helps individuals reduce their defenses and integrate a broader range of self-relevant information (Thomas et al., 2013). This process fosters a stable and coherent self-concept, enabling individuals to better understand their needs, values, and goals. Consequently, they are more likely to respond to negative emotions and stress adaptively, rather than resorting to avoidance behaviors like excessive phone use. In contrast, individuals with low self-compassion often lack a clear and consistent self-concept, which makes them more likely to perceive their surroundings as chaotic and unpredictable (Lee-Flynn, 2011), leading to increased negative emotions. Furthermore, individuals with low self-concept clarity struggle to process self-relevant information and use it to guide their behavior (Setterlund & Niedenthal, 1993). As a result, when confronted with stressful situations, they struggle to accurately describe and understand their experiences, weakening their emotional regulation abilities and leading them to rely more on experiential avoidance as a coping strategy. Given the convenience of smartphones, which become a primary tool for avoidance, they thereby exacerbate their smartphone addiction.

## 5. Implications and Limitations

These findings emphasize the importance of self-compassion in personal development and highlight its protective role against smartphone addiction tendency among college students. In addition to examining the direct effects of self-compassion, this study also investigates the mediating roles of self-concept clarity and experiential avoidance, which helps to reveal the multidimensional mechanisms of smartphone addiction tendency.

The results suggest that self-compassion enhances self-concept clarity, reduces the use of experiential avoidance as a coping strategy, and decreases the tendency toward smartphone addiction. These findings support and extend the social mentality theory of self-compassion, indicating that self-compassion is not only beneficial for mental health but also plays a positive role in preventing smartphone addiction tendency. Additionally, this study explores the causes of smartphone addiction tendency from the perspectives of individual traits (self-concept clarity) and coping strategies (experiential avoidance), supporting the I-PACE model.

The practical implications of this research are significant for preventing and intervening in smartphone addiction tendency among college students. Implementing self-compassion interventions could help students develop a clearer, more accurate self-concept, which in turn could enhance their emotional awareness and foster healthy coping strategies, ultimately reducing the smartphone addiction tendency. Research by Dundas et al. (2017) demonstrated that even brief self-compassion interventions could significantly improve self-regulation in university students, thereby enhancing their emotional management and behavioral responses. Therefore, universities could consider embedding brief mindfulness–compassion modules within campus counseling services, workshops, or online platforms as a widespread and sustainable intervention strategy. Furthermore, since experiential avoidance is a proximal risk factor for smartphone addiction, it could be addressed through psychological interventions, such as Acceptance and Commitment Therapy (ACT). These approaches could help students use smartphones more rationally and better manage the challenges posed by technological advancements.

Several limitations of this study should also be noted. First, this study’s reliance on cross-sectional self-reported data limits the causal inferences between self-compassion and smartphone use. Future research should use longitudinal designs or experimental methods to manipulate self-compassion levels and assess their effects on smartphone use. Additionally, combining objective measures, such as smartphone tracking apps with self-reports, would reduce the social desirability bias and improve data accuracy. Second, this study controlled for demographic variables alone, without considering other potential confounders, such as depressive symptoms. Previous research has indicated that depressive symptoms are closely associated with problematic smartphone use and may influence the relationship between self-compassion and smartphone use (Zhang & Wang, 2024). Future studies should consider including depressive symptoms and other relevant psychological factors to obtain a more comprehensive understanding of these relationships. Third, the sample was limited to college students; future research should include a broader, more diverse population to test whether these findings are generalizable across different groups. Lastly, the self is shaped by cultural influences (Heine, 2001), and the expression and impact of self-compassion vary across cultures (Arimitsu, 2023; Lou et al., 2022; Montero-Marin et al., 2018; Zhao et al., 2021). In China, individuals tend to incorporate constructive self-criticism and self-reflection into their understanding of self-compassion, reflecting a more dialectical perspective (Lou et al., 2022; Zhao et al., 2021). Given these cultural differences, it is essential to further explore and validate this model in various cultural contexts.

## 6. Conclusions

Through exploring the significant factors that may predict smartphone addiction tendency and their underlying mechanisms, this study finds that self-compassion not only directly and negatively predicts smartphone addiction tendency, but also indirectly predicts smartphone addiction tendency through the independent mediating effects of self-concept clarity and experiential avoidance, as well as the chain-mediating effect of self-concept clarity and experiential avoidance. Accordingly, by enhancing self-compassion, promoting self-concept clarity, and reducing experiential avoidance, smartphone addiction tendency among college students could potentially be reduced.

## Figures and Tables

**Table 1:** Discriminant validity of measurement tools.

Model		χ^2^	*df*	χ^2^/*df*	CFI	TLI	RMSEA	SRMR
M1	SC; SCC; EA; SAT	537.05	113	4.75	0.94	0.92	0.07	0.05
M2	SC + SCC; EA; SAT	860.15	116	7.42	0.89	0.87	0.10	0.06
M3	SC + SCC + EA; SAT	999.12	118	8.47	0.87	0.85	0.11	0.07
M4	SC + SCC + EA + SAT	1841.97	119	15.48	0.74	0.71	0.15	0.08

Note: Self-compassion (SC), self-concept clarity (SCC), experiential avoidance (EA), and smartphone addiction tendency (SAT); these are the same below.

**Table 2:** Descriptive statistics and partial correlation analysis of variables (n = 641).

Variables	M	SD	1	2	3	4
1. SC	3.36	0.61	1			
2. SCC	2.89	0.67	0.39 ***	1		
3. EA	3.73	1.28	−0.54 ***	−0.64 ***	1	
4. SAT	2.76	0.83	−0.42 ***	−0.51 ***	0.59 ***	1

Note: *** *p* < 0.001.

**Table 3:** Bias-corrected bootstrap test of the mediating effects.

Path	Standardized Effect Size	BootLLCI	BootULCI	Relative Mediation Effect
Total indirect effect	−0.33	−0.44	−0.25	65.80%
Path1: SC→SCC→SAT	−0.09	−0.16	−0.03	17.69%
Path2: SC→EA→SAT	−0.15	−0.24	−0.09	30.61%
Path3: SC→SCC→EA→SAT	−0.09	−0.14	−0.05	17.50%
Total effect	−0.50			

## Data Availability

The datasets processed and analyzed during the current study are available from the corresponding/first author upon reasonable request. The data are not publicly available due to their containing information that may compromise the participants’ privacy.
